# Behavioral Dynamics Analysis in Language Education: Generative State Transitions and Attention Mechanisms

**DOI:** 10.3390/bs15030326

**Published:** 2025-03-06

**Authors:** Qi Zhang, Yiming Qian, Shumiao Gao, Yufei Liu, Xinyu Shen, Qing Jiang

**Affiliations:** 1 School of Foreign Languages, Beijing Institute of Technology, Beijing 100081, China; 2 China Agricultural University, Beijing 100083, China; 3 College of Physical Education and Sports, Beijing Normal University, Beijing 100091, China; 4 Faculty of Arts and Sciences, Beijing Normal University, Zhuhai 519087, China

**Keywords:** behavioral dynamics modeling, adaptive learning path design, dynamic learning behavior prediction, generative attention mechanism, state transition equation

## Abstract

This study proposes a novel approach for analyzing learning behaviors in Chinese language education by integrating generative attention mechanisms and generative state transition equations. This method dynamically adjusts attention weights and models real-time changes in students’ emotional and behavioral states, addressing key limitations of existing approaches. A central innovation is the introduction of a generative loss function, which jointly optimizes sentiment prediction and behavior analysis, enhancing the adaptability of the model to diverse learning scenarios. This study is based on empirical experiments involving student behavior tracking, sentiment analysis, and personalized learning path modeling. Experimental results demonstrate this method’s effectiveness, achieving an accuracy of 90.6%, recall of 88.4%, precision of 89.3%, and F1-score of 88.8% in behavioral prediction tasks. Furthermore, this approach attains a learning satisfaction score of 89.2 with a 94.3% positive feedback rate, significantly outperforming benchmark models such as BERT, GPT-3, and T5. These findings validate the practical applicability and robustness of the proposed method, offering a structured framework for personalized teaching optimization and dynamic behavior modeling in Chinese language education.

## 1. Introduction

In the context of rapid globalization and digitalization, the field of Chinese language learning has been continuously exploring innovative technologies to enhance the effectiveness of international Chinese education (Vu et al., 2022; T. Yu et al., 2023; Y.-T. Yu & Tsuei, 2023). In recent years, the application of digital cultural resources in global education has gradually increased, with educators discovering that digitizing cultural heritage through modern technology can effectively bridge the gap between classroom learning and real cultural experiences (Gulyamov et al., 2024; Lian & Xie, 2024; Wang & Reynolds, 2024). In international Chinese education, the analysis of student behavior plays an important role in the innovation of teaching methods (Mariani et al., 2022; Tlili et al., 2023). Traditional teaching methods are often rigid and struggle to fully stimulate students’ subjective initiative. However, by utilizing digital cultural resources, not only can the content of teaching be enriched, but it can also promote students’ cross-cultural understanding and improve classroom interactivity and participation (Elimadi et al., 2024; Guo et al., 2023; Roll et al., 2021).

Safarov et al. (2023) proposed a DNN method to more accurately generate candidates in electronic learning platforms facing an exponential increase in available online courses and learners. Empirical results confirm the potential of the proposed solution. In particular, the accuracy for Top-1 and Top-5 courses was 0.626 and 0.492, respectively. For 25 and 50 new learners, the cold start errors were 0.618 and 0.697, respectively. Barrett et al. (2023) used the technology acceptance model to investigate learners’ attitudes towards Mozilla Hubs (a multiuser VR learning environment) for learning Chinese as a foreign language. Data were collected through post-participation surveys measuring seven structures. Structural equation modeling was used to explore the relationships between these structures. With the maturation of AI technology, the education field has gradually started to introduce AI into the classroom to enhance teaching effectiveness and personalized learning experiences (Abu-Ghuwaleh & Saffaf, 2023; Lu et al., 2023; Ottenbreit-Leftwich et al., 2023). Uiphanit et al. (2020) aimed to develop a mobile AR application to enhance Chinese vocabulary learning for students majoring in Chinese as a main subject. Afterward, 3D models, illustrations, and sounds were created based on the content, and software design and implementation followed. After this development process, 10th-grade Chinese major students from Hongtong Province A School tested the application. The evaluation was performed using pretest and post-test data analyzed through paired sample *t*-tests with 95% confidence. The results of this study indicate that the application was an effective tool for improving students’ learning abilities. Chen et al. (2021) reported on the use of interactive spherical video-based virtual reality (ISV-VR) in Chinese descriptive writing, revealing seven conceptual categories, their distribution in teachers’ concepts, and their hierarchical relationships. Despite these advancements, existing AI-driven educational models still face significant challenges in adaptively capturing student behaviors and emotions during the learning process. Traditional models, such as pretrained transformers like BERT and GPT-3, rely on static attention mechanisms that fail to adjust dynamically based on students’ emotional fluctuations and engagement levels. Moreover, most current AI-based educational tools focus primarily on content delivery and assessment, rather than real-time adaptive behavioral analysis, limiting their ability to effectively support personalized learning interventions. Additionally, while sentiment analysis techniques have been incorporated into some educational systems, they often treat emotions as static states rather than dynamically evolving responses, leading to suboptimal learning experiences. These limitations highlight the need for a more adaptive and context-aware AI-driven approach that can model both behavioral patterns and emotional transitions in real time.

In this study, we introduce an interactive attention mechanism, which analyzes students’ behaviors and feedback during the digital cultural resources learning process to dynamically adjust teaching strategies, ensuring students’ engagement and focus throughout the learning process. This mechanism can overcome the limitations of traditional teaching methods, promoting Chinese language learners’ understanding of the Chinese language and culture in different cultural contexts. By integrating a generative attention mechanism and a generative state transition equation, our model effectively captures temporal dependencies in student behavior and dynamically adapts to emotional shifts, providing a more personalized and responsive learning environment. The innovations in this study are as follows:

1. Introduction of interactive attention mechanisms to optimize behavior analysis: This study applies interactive attention mechanisms to the field of Chinese learning behavior analysis, using deep learning models to capture students’ behavioral characteristics during digital cultural resource learning and dynamically adjust the system’s focus. This mechanism can flexibly allocate attention to the personalized needs of different learners, addressing the issue of real-time feedback.
2. Utilizing digital cultural heritage resources to enhance the learning experience: Unlike traditional classroom teaching, this study introduces digital cultural heritage resources into Chinese language learning. By using rich virtual cultural resources, it provides an immersive learning experience. This not only helps students more intuitively understand Chinese culture but also promotes cross-cultural communication and language application skills development.
3. Building a generative model for learning behavior prediction and feedback: This study innovatively constructs a generative learning behavior prediction model by collecting students’ learning logs and interaction data, predicting learning behaviors and emotional states. The model is optimized using a generative loss function, allowing the system to provide personalized learning path recommendations based on predictions.
4. Spatial state transition equations for dynamic emotional and behavioral capture: In order to better understand students’ emotional and behavioral changes during the learning process, this study designs a spatial state transition equation to model the transition of students’ learning states. This equation describes the evolution of students from one learning state to another.

## 2. Related Work

### 2.1. Research on Traditional Teaching Methods

With the deepening of globalization and the rapid development of technology, the field of Chinese language learning has gradually explored the application of new media technologies in teaching to improve teaching effectiveness and meet diverse learning needs (Bernacki et al., 2021; Munna & Kalam, 2021). In recent years, more and more scholars have attempted to apply digital resources to language teaching, particularly in areas such as digital textbooks, online courses, teaching websites, and resource libraries, with numerous academic achievements that have been validated and recognized (Ezzaim et al., 2024; Fan & Antle, 2020; Ouyang & Zhang, 2024). Chinese language education has a long history and has played an important role in the dissemination of basic knowledge, but this method has certain limitations in analyzing student behavior and assessing learning effectiveness.

Traditional Chinese language teaching methods generally rely on teacher-led classroom instruction, emphasizing fixed textbooks and standardized assessment methods, with a focus on the transmission of knowledge points and mastery of language structures (Camacho-Morles et al., 2021). These traditional teaching methods often concentrate the evaluation of learning behaviors on exam scores and classroom performance, lacking dynamic tracking of the learning process (Kalogiannakis et al., 2021; Meddah & Benamara, 2024). In recent years, several emerging teaching tools have been applied to Chinese language learning. As teaching materials with rich content and interesting characteristics, digital cultural heritage resources cover cultural relics, human landscapes, artworks, and monumental buildings, which are stored and displayed in digital form through technology (Hein, 1991; Sweller, 2011). These resources not only have intuitiveness and immersiveness but also help students better understand the deeper meanings of Chinese culture while learning the language. When used properly in the classroom, these resources can effectively stimulate students’ interest in learning, create a relaxed and enjoyable learning atmosphere, enhance student participation, and play an important role in cross-cultural communication. However, despite the high application potential of digital cultural resources in education, research on their use in international Chinese education remains in the early exploratory stages. Existing studies have shown that current research mostly focuses on the digital protection and restoration of cultural heritage resources and information collection and storage, as well as resource display and dissemination, but the specific effectiveness and teaching model innovation in educational applications still need to be further explored (Alenezi, 2023).

Recent Chinese teaching research has started to focus on the teaching application of digital cultural resources, attempting to make Chinese language teaching more attractive and interactive by utilizing new media and digital technology. However, in these studies, teaching methods are often based on qualitative analysis and lack specific behavioral science evaluation tools. For example, many studies rely solely on qualitative observation or collect student feedback through teacher reports without using behavioral analysis techniques to quantitatively assess students’ learning behaviors and emotional states (Bilyalova et al., 2020; Selwyn, 2024). Since Chinese language learning is a long-term process of cognitive and cultural acquisition, the limitations of qualitative research make it difficult to accurately assess the deep impact of digital resources on student behavior and learning effectiveness.

### 2.2. Current Applications of Artificial Intelligence in Education

The application of Artificial Intelligence (AI) technology in education has been gradually increasing, particularly in areas such as personalized learning, student behavior analysis, and learning process evaluation, showing significant potential (Sapci & Sapci, 2020). Through technologies like natural language processing (NLP), deep learning, and machine learning, AI systems can extract valuable behavioral patterns and learning characteristics from vast amounts of learning data, thus providing technical support for personalizing and refining teaching methods (Chiu & Chai, 2020; Sanusi et al., 2024; W. Yang, 2022). In personalized learning, AI can recommend suitable learning content or adjust learning paths based on students’ learning progress and knowledge mastery, optimizing students’ learning outcomes. In particular, in language learning, AI, through real-time feedback mechanisms and personalized recommendation systems, enables students to perform more efficient language training, thereby enhancing both learning effectiveness and experience (Wong et al., 2020).

In student behavior analysis, AI technology uses machine learning algorithms to model students’ learning processes and predict their learning states based on behavioral data. For example, by analyzing students’ learning logs, online interaction frequency, and assignment completion, AI models can predict students’ emotional states (García-Martínez et al., 2023). Suppose the student behavior data are $X=\{x_{1},x_{2},...,x_{n}\}$, where $x_{i}$ represents the student’s behavioral feature at the *i*-th learning task. The AI model constructs a mapping function f:X→Y, where *Y* is the set of emotional states, and optimizes the mapping relation *f* through deep learning models to improve the accuracy of emotional state prediction. In the process of optimizing student behavior data mapping, deep learning models commonly use gradient descent to minimize the loss function between the predicted value and the actual value. In language education, the application of NLP technologies is particularly important. NLP techniques can analyze students’ language expressions and reading comprehension abilities, helping teachers identify students’ learning weaknesses and provide targeted guidance (Guan et al., 2020). However, despite the immense potential of AI technology in education, there are still certain limitations, particularly in dynamically assessing students’ emotional states and learning motivation (Liu & Wang, 2021).

### 2.3. AI-Based Student Behavior Analysis

Modern education emphasizes personalized learning experiences, and AI technology, through analyzing and predicting students’ behavior data, can significantly improve personalized recommendations during the teaching process, particularly in language education, where AI behavior analysis has a wide application (Bernardes et al., 2021). The key to behavior analysis is capturing students’ emotional changes and behavior patterns during the learning process so that teachers can adjust teaching strategies in real time according to students’ dynamic needs. Through multidimensional analysis of behavioral data, AI can quantify and predict students’ learning status, providing technical support for personalized teaching (Li et al., 2022).

In the field of Affective Computing, AI technology has gradually been applied to the recognition and analysis of students’ emotional states. Affective Computing is a technology that recognizes emotions based on multimodal features, such as facial expressions and voice tones, allowing real-time analysis of students’ emotional states (Marín-Morales et al., 2020). Suppose the emotional feature dataset of a student is $X=\{x_{1},x_{2},...,x_{n}\}$, where $x_{i}$ represents the emotional feature at the *i*-th time point, such as facial expression parameters or voice frequency characteristics. By constructing a mapping function f:X→Y, these features can be mapped to emotional states *Y*, such as “positive”, “neutral”, or “negative”. Through deep learning methods, *f* can be optimized to improve the accuracy of emotional state classification, allowing the AI model to provide support for adjusting teaching strategies based on students’ emotional changes. The loss function commonly used in emotional state prediction is cross-entropy loss, which measures the error between the predicted value $\hat{y}$ and the actual value *y*. The cross-entropy loss function *L* is defined as

(1) $$L\left(y,\hat{y}\right)=-\sum_{c=1}^{C}y_{c}\log\left({\hat{y}}_{c}\right)$$

where *C* is the number of emotional states, $y_{c}$ represents the actual category label, and ${\hat{y}}_{c}$ represents the predicted probability. By optimizing *L*, the model can gradually improve the accuracy of predicting students’ emotional states, thus providing reliable support for emotional analysis.

Generative AI techniques also have important applications in student behavior prediction, particularly in cases of sparse labeled data, where generative models can simulate students’ behavior paths and generate possible learning states to help teachers predict students’ learning emotions and participation. The generative model generates students’ behavioral features based on a probabilistic generative process. By maximizing the log-likelihood function, the generative model can generate virtual data that conforms to students’ behavioral patterns, providing a foundation for behavior prediction and personalized recommendation (Tahir et al., 2022). Future research could further develop AI systems based on emotional and behavioral feedback to better meet the needs of language education for personalized feedback and emotional support, thus improving teaching effectiveness.

## 3. Materials and Methods

### 3.1. Dataset Construction

#### 3.1.1. Source of Experimental Data

The data for this study come from multidimensional behavioral data and test scores, as well as interview feedback collected from international Chinese education students participating in the experiment. The experimental subjects are 14 first-year master’s students majoring in International Chinese Education (12 female, 2 male), aged between 21 and 23, from six countries: South Korea, Russia, Ghana, Vietnam, Pakistan, and Nigeria. These students are all Chinese learners with a high level of Chinese proficiency and some knowledge of Chinese culture. The main teaching content of the experiment focuses on the cultural knowledge of Chinese bronzeware and porcelain, with 10 class hours dedicated to explaining aspects of bronzeware and porcelain such as decorations, inscriptions, and forms. The course design aims to enhance students’ understanding of bronzeware and porcelain and foster their interest and identification with Chinese culture.

The primary tools used in data collection include VR headsets, 3D artifact models, and PowerPoint presentations. These diverse digital resources were used to collect behavioral data and learning feedback from students in the classroom from multiple perspectives and levels. The data collection is not limited to classroom performance but also includes pretest and post-test scores and interview feedback, among other sources. To further evaluate the effectiveness of digital cultural and museum resources in cultural classrooms, this study uses empirical experiments to collect students’ learning outcomes and behavioral data under different teaching conditions. The research team divides the experimental subjects into two groups, one using traditional teaching methods and the other using digital cultural resources assisted by the interactive attention mechanism, to explore the role of digital resources in improving learning outcomes. This design allows for direct comparative analysis, minimizing inter-individual variance and ensuring that results are statistically meaningful despite the smaller sample size. Furthermore, to enrich the data sources, interviews were conducted to capture students’ subjective experiences with the digital cultural resources and their feedback on the teaching process, providing deeper insights into the practical application of these resources in Chinese cultural education. The multisource data provide detailed research evidence for this study, validating the effectiveness of the interactive attention mechanism supported by multidimensional data.

#### 3.1.2. Data Collection Process

The data collection process in this study consists of three stages: pretest, classroom experiment, and post-test. First, to ensure that the experimental subjects have comparable initial learning levels, the research team conducted a pretest before the start of the teaching. The pretest mainly consisted of 40 multiple-choice questions and 4 subjective short-answer questions, with a total score of 50 points. The content covered the basic knowledge of bronzeware and porcelain and their cultural and historical background. Through normality testing and *t*-tests on the pretest scores, the results indicate that there were no significant differences in the initial learning levels between the experimental group and the control group, ensuring homogeneity at the beginning of the experiment.

Next, data collection during the classroom experiment was conducted. Over the course of five weeks, the research team rigorously controlled the teaching environment to ensure that the only difference between the experimental and control groups was the teaching method. Specifically, the experiment was divided into two themes: “Chinese Ritual Bronzes” and “Chinese Porcelain”. In the first theme, students in the experimental group learned bronzeware by observing 3D models through VR devices, while the control group used traditional PPT slides to view images of bronzeware. In the second theme, the teaching methods were switched, with the experimental group observing 3D porcelain models through VR devices, while the control group continued with traditional teaching methods. During the data collection process, the research team not only recorded students’ interaction behaviors and learning feedback during the VR model observations but also analyzed students’ learning engagement and behavioral performance through classroom recordings, in order to understand the specific impact of the interactive attention mechanism on the classroom learning process.

To more comprehensively evaluate the role of digital cultural resources in classroom teaching, the third stage of data collection, the post-test, further verified the persistence and effectiveness of the teaching outcomes. The post-test consisted of two phases: an immediate test and a delayed test. The first post-test was conducted immediately after the course, and the second delayed post-test was conducted three months later to assess the long-term retention of knowledge about bronzeware and porcelain. Similar to the pretest, the post-test included 40 multiple-choice questions and 5 subjective short-answer questions, with a total of 50 points, but the questions were modified to reflect the course content. We used SPSS 30.0.0 for the relevant statistical tests. Pretest scores were used to verify whether the learning levels of the two groups of participants were consistent. Post-test scores were used to validate the hypothesis that digital cultural resources can enhance the teaching effectiveness of international Chinese education practitioners, help participants better absorb and understand the relevant cultural content, and improve their academic performance. The test results are shown in Table 1, Table 2, Table 3 and Table 4:

Table 1 shows that the average scores of Group A and Group B are the same, both being 35.143. According to the results of the Kolmogorov–Smirnov (KS) test and Shapiro–Wilk (SW) test, the pretest scores of both groups exhibit normality characteristics. Table 2 presents the *t*-test results of the pretest scores, with *p*-values equal to 1, indicating no significant difference between the two groups. This suggests that the two groups had similar levels of understanding of the bronze and porcelain themes before the experiment, meeting the homogeneity requirement of the experiment.

From Table 3, it can be observed that the average scores of Group A and Group B for basic knowledge of bronze and porcelain artifacts, bronze-themed knowledge, and porcelain-themed knowledge are (9.714, 8.571), (15.143, 17.571), and (16.714, 14.429), respectively. The differences in average scores for the three types of knowledge are 1.143, 2.428, and 2.285, respectively, indicating that the differences in basic knowledge of bronze and porcelain artifacts are minor. Group B outperformed Group A in the bronze-themed knowledge, while Group A outperformed Group B in the porcelain-themed knowledge. Regardless of the knowledge type, the normality test results for both groups indicate that all scores exhibit normality characteristics. Table 4 shows that the scores for all three knowledge categories are significantly different. For basic knowledge of bronze and porcelain, the *p*-value is 0.347, indicating no significant difference between the two groups. However, for the bronze-themed and porcelain-themed scores, there are significant differences between the two groups, with *p*-values of 0.033 and 0.032, respectively, both less than 0.05.

From the above results, it can be concluded that the experimental group using 3D models for learning achieved higher accuracy and better performance when answering relevant questions. In contrast, the group that did not use or learn with 3D models exhibited lower accuracy and poorer performance. Compared with traditional teaching methods, using digital cultural resources to assist cultural teaching helps improve students’ scores, deepen their understanding and retention of cultural knowledge, and enhance the overall effectiveness of cultural education.

Additionally, this study also conducted interviews to collect students’ subjective experiences with digital cultural resources to further verify the practical effectiveness of the interactive attention mechanism in the classroom. Four students were randomly selected from the experimental and control groups for semi-structured interviews, with six core questions designed to cover aspects such as the intuitive experience of digital cultural resources, learning efficiency, classroom participation, and the difficulty of using the equipment. The specific interview information is shown in Table 5.

All interviewed students agreed that VR and 3D artifact models significantly improved the intuitiveness and enjoyment of learning, enhanced their understanding and memory of cultural knowledge, and increased participation in the classroom. Some respondents mentioned that there were still some technical difficulties in operating the digital resources and that the equipment requirements were relatively high, suggesting that for broader applications in teaching, schools would need to support the necessary hardware. The interview data, as a supplement to the classroom experimental data, further demonstrate the significant effect of the interactive attention mechanism in improving students’ learning behaviors.

The data collection process of this study is centered around empirical experiments and supplemented by interview data. By validating the application of the interactive attention mechanism in Chinese learning behavior analysis from multiple data angles, the diverse data sources and the rigorous data collection process ensure the reliability and scientific accuracy of the research findings.

#### 3.1.3. Ethical Considerations and Data Source Compliance

The data used in this study were collected from teaching experiments conducted at the Beijing Institute of Technology, ensuring full compliance with ethical guidelines. This study did not involve any procedures that could cause physical or psychological harm to participants, nor did it involve sensitive personal information or commercial interests. According to the Medical and Experimental Animal Ethics Committee of the Beijing Institute of Technology, research that does not pose risks to human subjects and utilizes anonymized educational data is exempt from ethical review. All participants were fully informed about the study’s objectives and voluntarily participated. Additionally, data collection and processing followed strict anonymization protocols, ensuring that no personally identifiable information was stored or analyzed. These measures align with institutional ethical policies, ensuring that the study adheres to responsible data usage and research integrity standards.

#### 3.1.4. Data Processing and Feature Extraction

In this study, data processing and feature extraction mainly include data cleaning, feature extraction, and data normalization and standardization. These processes provide high-quality input for the modeling of the interactive attention mechanism, allowing for more accurate analysis of students’ learning behaviors and emotional changes. Data cleaning is the first step in data preprocessing, aiming to handle missing values, outliers, and noise in the data to ensure the integrity and quality of the data. During data collection, students’ learning behavior data may include issues such as incomplete submissions or incorrect clicks. If these problems are not addressed, they may affect the accuracy of the experimental results. Therefore, for missing values, interpolation methods or mean imputation strategies can be used. For example, if student *i* has missing learning duration data, and the average learning duration of other students is $\bar{x}$, the missing value can be filled with $\bar{x}$, defined as

(2) $$x_{i}=\left\{\begin{array}{cc} \bar{x} & \mathrm{if}\;x_{i}\;\mathrm{is}\;\mathrm{missing}\\ x_{i} & \mathrm{if}\;x_{i}\;\mathrm{is}\;\mathrm{not}\;\mathrm{missing} \end{array}\right.$$

Outlier processing is typically based on statistical methods to detect data points that are far from the mean. For instance, in the case of student engagement data, if a data point deviates from the mean by more than twice the standard deviation, it is considered an outlier. If $x_{i}$ is the engagement level of student *i*, and the mean of the data is μ with a standard deviation of σ, then data points satisfying the following condition are considered outliers and may be deleted or replaced:

(3) $$|x_{i}-\mu |>2\sigma$$

After data cleaning, feature extraction is performed to obtain valid learning features from students’ behavior data, providing a foundation for subsequent analysis and modeling. In this study, the main features extracted include student participation, emotional changes, the time distribution of learning behaviors, and learning outcomes and feedback, which reflect students’ learning processes from different perspectives. Participation is an important indicator to assess student engagement in learning, such as the number of discussions participated in and the frequency of questions asked. If student *i* has $d_{i}$ discussion attempts and $q_{i}$ question frequency during the experiment, the total participation $E_{i}$ can be calculated as a linear combination:

(4) $$E_{i}=\alpha d_{i}+\beta q_{i}$$

where α and β are weight parameters that reflect the relative importance of discussions and questions in the participation measure. Emotional changes are used to analyze the fluctuations in students’ emotions during the learning process, and emotional states are extracted using affective computing techniques. Suppose during the learning process, the emotional state $y_{i}$ of a student can take values such as “positive”, “neutral”, or “negative”, and can be predicted using an emotional classification model f(x):

(5) $$y_{i}=f\left(x_{i}\right)$$

The time distribution of learning behavior is an important feature for analyzing students’ learning rhythm, such as the duration and intervals between each learning session. By conducting a time series analysis, students’ learning behavior patterns during different periods can be captured. Suppose the learning durations of student *i* at different time points are $\left\{t_{1},t_{2},...,t_{n}\right\}$, the average learning time $\bar{t}$ can be calculated as

(6) $$\bar{t}=\frac{1}{n}\sum_{i=1}^{n}t_{i}$$

Learning outcomes and feedback features reflect the effectiveness of students’ learning through test scores, assignment grades, etc. Suppose student *i*’s scores in multiple tests are $\left\{s_{1},s_{2},...,s_{n}\right\}$, then the total learning outcome $S_{i}$ is the weighted sum of all test scores:

(7) $$S_{i}=\sum_{j=1}^{n}\gamma_{j}s_{j}$$

where $\gamma_{j}$ is the weight of the *j*-th test. After data cleaning and feature extraction, data normalization and standardization are crucial steps to ensure that each feature has a balanced impact on the model’s predictions. In this study, features may have different units or scales, and direct input into the model could lead to biased results. To eliminate the influence of units, data normalization can map each feature to the range [0,1]. For example, if the feature value range is $\left[x_{\min},x_{\max}\right]$, the normalized value $x^{\prime }$ is calculated as

(8) $$x^{\prime }=\frac{x-x_{\min}}{x_{\max}-x_{\min}}$$

If standardization is used, the feature values can be transformed to a standard normal distribution with a mean of 0 and a standard deviation of 1. Let the mean of the feature *x* be μ and the standard deviation be σ; then, the standardized feature value $x^{\prime \prime }$ is

(9) $$x^{\prime \prime }=\frac{x-\mu }{\sigma }$$

Standardized data eliminate scale differences between features, allowing for a more balanced impact on the model and helping to improve the accuracy and adaptability of the model in analyzing student behavior.

In this study, the selection and weighting of student behavior metrics, including participation, emotions, and test scores, were determined through a combination of teaching experience and data-driven feature importance analysis. Specifically, the selection of these features was guided by pedagogical relevance, ensuring that they effectively represent key aspects of student engagement and learning performance. To quantitatively assess their impact, Principal Component Analysis (PCA) was employed to evaluate the relative importance of each feature in predicting learning behaviors. The PCA results confirm that participation (e.g., discussion frequency, question attempts), emotional fluctuations, and learning outcomes are the most influential factors in student engagement modeling. Based on this analysis, feature weighting was adjusted accordingly. For instance, the contribution of participation metrics was fine-tuned by assigning appropriate weights (α,β) to discussion and questioning behaviors, while learning outcomes were aggregated using weighted summation to reflect their significance. This hybrid approach ensures that the model dynamically adapts to student behaviors while maintaining high predictive accuracy, ultimately enhancing its ability to provide personalized learning interventions.

### 3.2. Proposed Method

#### 3.2.1. Overview

**Figure 1:**
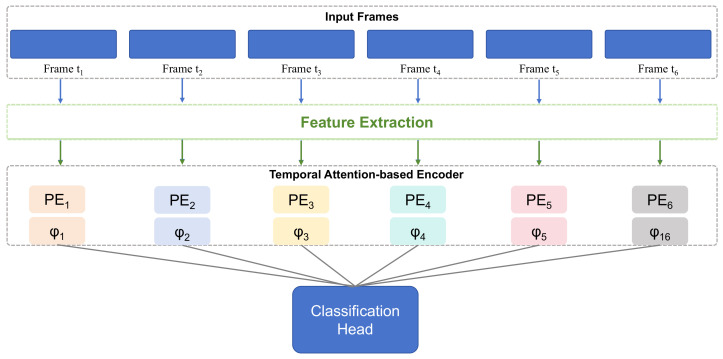
Flowchart of the proposed method.

This study proposes a method for Chinese learning behavior analysis based on an interactive attention mechanism, aiming to optimize teaching strategies in the Chinese learning process using digital cultural resources to enhance learning outcomes, as shown in Figure 1. The core idea of this method is to introduce generative attention mechanisms, generative state transition equations, and generative loss functions to analyze student learning behaviors and emotional changes in real time. This enables dynamic adjustments to teaching strategies and the provision of personalized learning paths. This approach not only captures the behavioral patterns of students during the learning process, but also predicts their emotional states and learning progress, ultimately achieving refined instructional interventions to improve student engagement, focus, and learning results.

The overall framework of this research includes four main components: generative attention mechanisms, generative state transition equations, generative loss functions, and model training and optimization. Each part integrates deep learning techniques and generative models to effectively handle dynamic changes and complex behaviors in students’ learning processes, providing new technical pathways for behavioral analysis in Chinese education.

#### 3.2.2. Generative Attention Mechanism

**Figure 2:**
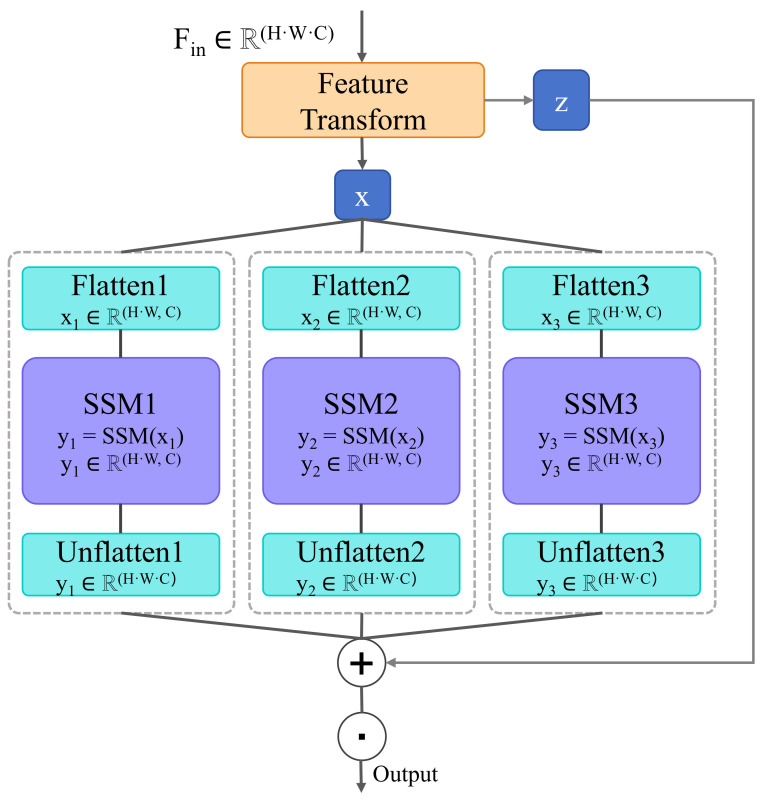
Generative attention mechanism.

The generative attention mechanism is one of the key technologies introduced in this study to address the limitations of traditional self-attention mechanisms in dynamic student learning behavior analysis, as shown in Figure 2. Self-attention is the core module in Transformer models, where it dynamically adjusts the input sequence representation by calculating the correlations between different elements of the input sequence. While self-attention has achieved significant success in many natural language processing tasks, it has certain limitations when processing complex and dynamic learning behaviors and emotional states. Therefore, this study introduces the generative attention mechanism, which transforms the allocation of attention from a static process to a dynamic one, generated based on real-time student learning behaviors, better adapting to the emotional fluctuations and behavioral changes in students during the learning process.

The core idea of the generative attention mechanism is to dynamically generate attention distributions through a generative model. Unlike the traditional fixed-weight computation, the generative attention mechanism generates dynamic attention weights based on features in the input sequence and historical student behavior data. This generative process relies on real-time student learning data, adjusting the model’s focus at each time step to better match the personalized needs of the student in the learning process. The design of the generative attention mechanism differs from traditional self-attention by incorporating a generative module, which generates attention weights at each time step by learning the underlying patterns in student historical behavior data. Specifically, we use generative models based on deep learning, such as Recurrent Neural Networks (RNNs) or Long Short-Term Memory Networks (LSTMs), combined with traditional attention mechanisms to generate attention distributions at each layer. The network structure of the module is as follows:

1. Input Layer: The input layer receives the behavior features of the student during the learning process, such as study duration, classroom participation, emotional states, etc. These features are passed through an embedding layer to map the raw input into a higher-dimensional feature space.
2. Generative Module: An LSTM layer is used to capture the temporal features of student behavior. The LSTM layer, through memory cells and gating mechanisms, can effectively capture long-term dependencies in students’ learning processes, thereby generating attention weights at each time step.
3. Attention Calculation Layer: The output of the generative module is used as a query vector, which is then used to compute the attention weights by performing attention operations with the key and value matrices from the input sequence.
4. Output Layer: The output layer generates the final weighted feature representation, which is used for subsequent learning behavior prediction or emotional state analysis.

The mathematical formulation of the generative attention mechanism can be divided into two parts: generating attention weights and computing the weighted feature representations. Generating attention weights: In traditional self-attention mechanisms, attention weights are statically assigned based on the similarity between input elements. In the generative attention mechanism, attention weights are generated dynamically based on student behavior data through a generative model, represented as

(10) $$w_{t}=g\left(f\left(S_{t-1},X_{t}\right)\right),$$

where $S_{t-1}$ is the student’s learning state at time step t−1, $X_{t}$ is the student’s behavior features at time step *t*, *f* represents the generative model (e.g., LSTM), *g* is the generative function, and $w_{t}$ is the generated attention weight. Weighted feature representation: The generated attention weight $w_{t}$ is multiplied with the value matrix $V_{t}$ from the input sequence to obtain the weighted feature representation:

(11) $${\mathrm{Output}}_{t}=w_{t}\cdot V_{t}$$

In this way, the generative attention mechanism can dynamically adjust the distribution of attention based on student learning behaviors and emotional changes. Compared with traditional self-attention mechanisms, the generative attention mechanism has significant advantages, especially in handling dynamic and complex learning behavior data, making it better suited to the personalized needs of students. The specific advantages are as follows:

1. Dynamic Adaptability: The generative attention mechanism dynamically adjusts attention weights based on the student’s current learning state, while traditional self-attention is static and cannot respond in real time to changes in student behavior. Students may experience different emotional fluctuations and behavioral changes during the learning process, such as declining interest or the onset of anxiety. The generative attention mechanism can adjust the attention distribution in real time based on these changes, providing more precise feedback.
2. Personalized Learning Path: By dynamically generating attention weights, the generative attention mechanism can customize the learning path for each student. As students’ behavioral patterns and emotional states change with the progression of learning, the generative mechanism can adjust learning content and teaching strategies accordingly, thereby improving student engagement and learning outcomes.
3. Long-Term Dependency Modeling: By incorporating generative models (such as LSTM), the generative attention mechanism can capture long-term dependencies in students’ learning processes, identifying their learning patterns and generating dynamic attention weights that adapt to these patterns. This is crucial for predicting long-term learning behaviors and capturing emotional fluctuations.
4. Improved Emotional and Behavioral Prediction Accuracy: Traditional self-attention mechanisms can capture short-term learning behaviors, but due to the lack of emotional fluctuation detection, the prediction results may not be accurate. The generative attention mechanism, by combining generative models and deep learning techniques, can more accurately identify changes in students’ emotional states, improving the accuracy of both emotional and behavioral predictions.

In conclusion, the generative attention mechanism offers a dynamic and adaptive way to allocate attention based on students’ real-time learning behaviors and emotional states. This method not only improves the accuracy of predicting learning behaviors but also enhances the model’s ability to provide personalized learning paths, making it more suitable for handling complex and dynamic student learning data. It allows for real-time adjustments to teaching strategies, providing more personalized learning support and thus improving overall learning outcomes.

#### 3.2.3. Generative State Transition Equation

**Figure 3:**
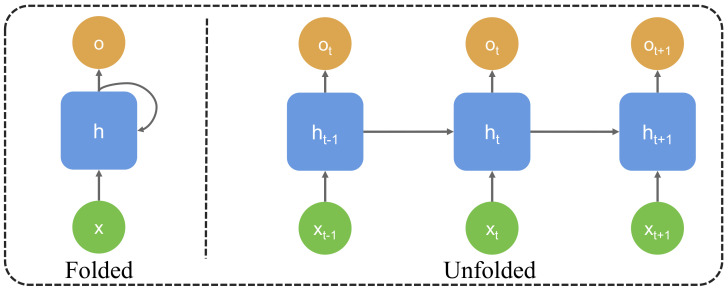
Generative state transition equation.

The design of the generative state transition equation in this paper aims to capture the dynamic changes in students’ learning processes, as shown in Figure 3. Under the framework of the generative attention mechanism, students’ learning behaviors and emotional states not only depend on current input features but are also closely related to their historical behaviors. The goal of the generative state transition equation is to establish a mathematical model that can describe the transition of students from one learning state to another and incorporate this transition into the model’s decision-making process. To better capture such state transitions, we adopt a method based on Markov Decision Processes (MDPs) to design the state transition equation.

Specifically, let the learning state of a student at time step *t* be represented by $S_{t}$. This state not only includes the student’s current behavioral features but also reflects multiple aspects such as emotional states and learning progress. We assume that the transition of the student’s state at a particular time step is determined by a generative process. In other words, the student’s current learning state $S_{t}$ is jointly determined by the previous learning state $S_{t-1}$, the current learning input features $X_{t}$, and the generated action $A_{t}$. The mathematical expression of the generative state transition equation is as follows:

(12) $$S_{t}=f\left(S_{t-1},A_{t},X_{t}\right)+\epsilon_{t}$$

where *f* represents a generative function that learns the patterns from the student’s historical data to generate the current learning state. $A_{t}$ represents the teaching action taken at time step *t* (such as adjustments to course content or teaching methods), $X_{t}$ represents the student’s learning behavior features at time step *t*, and $\epsilon_{t}$ is the noise term that accounts for external factors or random changes in the learning process.

This design is based on a generative model. By considering the student’s behavior and emotional changes throughout the historical learning process, it captures the dynamic transitions of students at different learning stages. The generative function *f* learns the long-term dependencies of the student through deep learning models (such as LSTM or Transformer) to predict the current learning state. By combining $S_{t}$ with the historical state $S_{t-1}$, the current behavior $X_{t}$, and the teaching action $A_{t}$, the model can generate new learning states at each time step based on the student’s learning behavior and emotional state, thereby providing dynamic feedback for teaching intervention. In specific terms, the generative state transition equation provides the process of a student transitioning from one learning state to another, while the generative attention mechanism plays a key role in adjusting the teaching intervention during this process. When the student’s learning state changes, the generative state transition equation can predict the student’s current state based on historical data and provide guidance for selecting the next action. In this process, the generative attention mechanism dynamically adjusts the allocation of attention according to the student’s current emotional state and learning state to make teaching interventions more precise. For example, when the student experiences anxiety or emotional downturns, the generative state transition equation can predict the impact of these emotions on the student’s learning state. The generative attention mechanism, in turn, adjusts the student’s learning progress accordingly and fine-tunes teaching strategies, helping students regain interest and motivation in their studies.

#### 3.2.4. Generative Loss Function

In this study, the generative loss function plays a crucial role in optimizing the generative attention mechanism and state transition equations. Traditional loss functions are typically fixed and designed with explicit objectives based on specific tasks, such as the mean squared error (MSE) loss for regression problems and the cross-entropy loss for classification problems. The MSE loss primarily measures the difference between predicted values and true values, and the model is optimized by minimizing this loss function:

(13) $$L_{\mathrm{MSE}}=\frac{1}{N}\sum_{i=1}^{N}{\left(y_{i}-{\hat{y}}_{i}\right)}^{2}$$

where $y_{i}$ represents the true value, ${\hat{y}}_{i}$ is the model’s predicted value, and *N* is the number of samples. The cross-entropy loss is applied in classification tasks to measure the difference between the predicted probability distribution of classes and the true class labels:

(14) $$L_{\mathrm{CE}}=-\sum_{c=1}^{C}y_{c}\log\left({\hat{y}}_{c}\right)$$

where $y_{c}$ represents the true label for class *c*, ${\hat{y}}_{c}$ is the predicted probability of class *c*, and *C* is the number of classes. Unlike traditional loss functions that focus on a single objective, the generative loss function introduced in this study simultaneously optimizes behavior prediction and emotion recognition, allowing the model to integrate both aspects into a holistic understanding of the learning process. This function helps the model adjust attention weight distributions over time, enabling it to adapt dynamically to changes in student behavior and emotional state transitions. The generative loss function consists of two components: the behavior prediction loss and the emotion prediction loss. The behavior prediction loss measures the discrepancy between the predicted and true student behaviors, while the emotion prediction loss ensures accurate classification of emotional states. The mathematical expression of the generative loss function is

(15) $$L\left(\theta \right)=\lambda_{1}L_{\mathrm{behavior}}\left(\theta \right)+\lambda_{2}L_{\mathrm{emotion}}\left(\theta \right)$$

where $L_{\mathrm{behavior}}\left(\theta \right)$ represents the loss for student behavior prediction, $L_{\mathrm{emotion}}\left(\theta \right)$ represents the loss for emotion prediction, and $\lambda_{1}$ and $\lambda_{2}$ are weight coefficients used to balance the optimization objectives for behavior and emotion predictions. The behavior prediction loss $L_{\mathrm{behavior}}\left(\theta \right)$ is typically calculated using the mean squared error (MSE), which measures the discrepancy between the predicted student behavior and the true value. In this task, the predicted student behavior can include factors like learning time, participation, and learning progress. The MSE loss can be used to calculate the difference between the predicted values and actual observations of these factors. The mathematical expression for the behavior prediction loss is

(16) $$L_{\mathrm{behavior}}\left(\theta \right)=\frac{1}{N}\sum_{i=1}^{N}{\left(y_{i}-{\hat{y}}_{i}\right)}^{2}$$

where $y_{i}$ represents the true behavior state of the *i*th student (e.g., learning time, participation), ${\hat{y}}_{i}$ is the model’s predicted behavior state, and *N* is the number of samples. The emotion prediction loss $L_{\mathrm{emotion}}\left(\theta \right)$ is calculated using cross-entropy loss, which measures the prediction error for emotional state classification. During the learning process, a student’s emotional state (e.g., anxiety, excitement, boredom) can affect their learning behavior, especially when facing challenging tasks. Therefore, the accuracy of emotion analysis is crucial for predicting learning behavior. The loss for emotion prediction can be defined as

(17) $$L_{\mathrm{emotion}}\left(\theta \right)=-\sum_{c=1}^{C}y_{c}\log\left({\hat{y}}_{c}\right)$$

where $y_{c}$ represents the true label for emotion class *c*, ${\hat{y}}_{c}$ is the predicted probability of class *c*, and *C* is the number of emotion classes. To optimize the generative loss function, gradient descent is used to minimize L(θ). Specifically, the optimization process involves computing the gradient of the loss function with respect to the model parameters θ. The gradient descent update rule is given by

(18) $$\theta_{t+1}=\theta_{t}-\eta \nabla_{\theta }L\left(\theta \right)$$

where η is the learning rate, and $\nabla_{\theta }L\left(\theta \right)$ represents the gradient of the loss function with respect to the model parameters θ. During training, the model iteratively updates its parameters θ to minimize the generative loss function, improving the accuracy of predictions for both student behavior and emotional states.

The generative loss function plays a crucial role in ensuring the model’s adaptability and responsiveness in student behavior prediction. Unlike traditional static models, which rely on fixed patterns, the generative loss function enables the model to continuously update its understanding of student engagement. This dynamic adaptation allows for real-time adjustments to teaching interventions, ensuring that the learning process remains responsive to students’ evolving needs. Another significant impact of the generative loss function is its ability to integrate behavioral and emotional aspects. Since learning behaviors are inherently influenced by emotional states, the model is designed to holistically predict both dimensions, leading to more accurate learning state estimations. By incorporating emotional state recognition into behavior prediction, the model provides personalized feedback that aligns with the student’s cognitive and emotional conditions, ultimately enhancing the learning experience.

Additionally, the generative loss function contributes to a time-aware attention mechanism. The model dynamically reallocates attention weights in response to fluctuations in student engagement, allowing it to prioritize the most relevant learning behaviors and emotional cues at different time steps. This ensures that the model remains sensitive to variations in student participation and emotional shifts, making it more effective in adaptive learning scenarios. By integrating these elements, the generative loss function provides a robust framework for real-time behavior prediction and intervention, making it a fundamental component of adaptive, student-centered learning environments.

#### 3.2.5. Model Training and Optimization

The training and optimization of the model are key steps to ensure the effective application of the generative attention mechanism and state transition equations. To optimize the generative model, this study uses gradient descent-based optimization algorithms, such as the Adam optimizer, to minimize the loss function L(θ). Specifically, the model parameters θ are updated according to the following formula:

(19) $$\theta_{t+1}=\theta_{t}-\eta \nabla_{\theta }L\left(\theta \right)$$

where η is the learning rate, and $\nabla_{\theta }L\left(\theta \right)$ represents the gradient of the loss function with respect to the model parameters θ. During training, the model continuously updates its parameters based on the student’s historical behavior data, emotional changes, and learning progress, in order to minimize the loss function, thereby improving the prediction accuracy and adaptability of the model. To avoid overfitting, early stopping and regularization techniques are employed during the training process. Early stopping monitors the loss function value on the validation set, and if the loss does not decrease further, the training is stopped early, thus preventing the model from overfitting to the training set. Regularization techniques, such as L2 regularization, add a penalty term to the loss function to limit the excessive fluctuation of model parameters and improve the model’s generalization ability. Once the model training is completed, cross-validation is used to further evaluate the model’s performance, ensuring its stability and reliability across different datasets. Through the training and optimization process outlined above, the generative attention mechanism model can effectively capture students’ learning behaviors and emotional changes, providing real-time feedback and personalized teaching interventions.

## 4. Results

### 4.1. Experimental Setup and Comparison Models

#### 4.1.1. Software and Hardware Configuration

This study utilized a high-performance computing server for hardware, equipped with dual Intel Xeon Gold 6248R processors, each featuring 24 physical cores and a base clock speed of 3.0 GHz. The server was supported by 512 GB of DDR4 memory, ensuring sufficient capacity for large-scale data processing and model training. To meet the computational demands of deep learning, the server was further equipped with four NVIDIA Tesla V100 GPUs, each with 32 GB of memory, totaling 128 GB of GPU memory. These high-performance GPUs significantly accelerated the training and inference processes of the deep learning models.

For the software environment, the operating system was Ubuntu 20.04 LTS, known for its stability and robust support for various deep learning frameworks. The deep learning framework used was PyTorch 1.8, combined with CUDA 11.1 and cuDNN 8.0, to fully leverage GPU computing capabilities. NVIDIA’s NCCL library and PyTorch’s DistributedDataParallel module were employed for efficient distributed training across multiple GPUs. Data processing and analysis were carried out using Python 3.8, with scientific computing libraries such as NumPy and Pandas used for data preprocessing. For database management, MySQL 8.0 was used to store large volumes of experimental data, including student behavioral data, emotional state labels, and intermediate model results.

#### 4.1.2. Comparison Models and Parameter Settings

In the model training process, the learning rate η was set to $1\times 10^{-4}$, and the Adam optimizer was adopted with $\beta_{1}$ and $\beta_{2}$ set to 0.9 and 0.999, respectively, to balance convergence speed and stability. The batch size was chosen as 64 to accommodate GPU memory constraints while maintaining training efficiency. The maximum number of training epochs was set to 50, with early stopping strategies employed to avoid overfitting. Regularization was applied through a weight decay factor of $1\times 10^{-5}$ and a dropout rate of 0.1, further enhancing model generalization. These hyperparameter settings were fine-tuned over multiple experiments to ensure the model’s efficiency and robustness in sentiment analysis and behavioral prediction tasks.

To validate the proposed method, several representative baseline models were selected for comparison, including BERT (Devlin, 2018), LLAMA (Touvron et al., 2023), RoBERTa (Liu et al., 2019), GPT-3 (Floridi & Chiriatti, 2020), XLNet (Z. Yang et al., 2019), T5 (Raffel et al., 2020), and ELECTRA (Clark et al., 2020). These models span the main natural language processing methodologies and pretraining approaches, providing a comprehensive evaluation of performance in sentiment analysis and behavioral dynamics capture tasks. BERT, as a bidirectional encoder pretraining model, captures contextual features through masked language modeling with its multilayer Transformer structure, demonstrating notable language modeling capabilities but limited by the static nature of its attention mechanism in dynamic sequential data. LLAMA, a lightweight generative model, is parameter-efficient and suitable for high-speed inference but underperforms in complex tasks compared with large-scale models. RoBERTa improves BERT’s training strategies by increasing batch sizes and removing the next sentence prediction task, enhancing language modeling performance but showing limited efficacy in behavioral dynamic modeling. GPT-3, as a large-scale generative pretraining model, excels in contextual understanding tasks but lacks adaptability in dynamically adjusting personalized learning paths. XLNet addresses BERT’s bidirectionality limitations through autoregressive modeling but struggles with complexity in capturing long-term dependencies. T5 unifies text generation frameworks for multitask learning, demonstrating superior performance in sentiment analysis and behavioral prediction but remains limited in handling dynamic transitions. ELECTRA, with its generator–discriminator architecture, significantly improves training efficiency and is effective in low-resource scenarios.

#### 4.1.3. Evaluation Metrics

In this experiment, multiple evaluation metrics were used to compare the performance of different models, including accuracy, precision and recall, F1-score, and AUC-ROC curve. These metrics provide a comprehensive way to assess the model’s ability to predict student learning behaviors and emotional states. Accuracy represents the proportion of correctly predicted samples in the predicted results and is used to evaluate the overall correctness of the model in predicting student behavior. Precision and recall reflect the model’s correctness and coverage in different emotional states and behavior prediction tasks, respectively. The F1-score combines precision and recall, providing a more comprehensive evaluation of the model’s performance. The AUC-ROC curve is used to assess the model’s ability to predict behavior at different learning stages, particularly its ability to distinguish between emotional categories. Let TPR represent the true positive rate and FPR represent the false positive rate, then the AUC value represents the integral area between TPR and FPR.

The formulas for each evaluation metric are as follows:

(20) $$\mathrm{Accuracy}=\frac{TP+TN}{TP+TN+FP+FN}$$

(21) $$\mathrm{Precision}=\frac{TP}{TP+FP}$$

(22) $$\mathrm{Recall}=\frac{TP}{TP+FN}$$

(23) $$F1-\mathrm{score}=2\cdot \frac{\mathrm{Precision}\cdot \mathrm{Recall}}{\mathrm{Precision}+\mathrm{Recall}}$$

where TP is the number of true positives (True Positive), TN is the number of true negatives (True Negative), FP is the number of false positives (False Positive), FN is the number of false negatives (False Negative), TPR is the true positive rate, FPR is the false positive rate. These metrics allow for a comprehensive evaluation of the model’s predictive performance, providing a more thorough assessment of the model’s effectiveness.

### 4.2. Behavior Prediction Results

The purpose of this experiment is to compare the performance of multiple models on the task of student behavior prediction and validate the superiority of the proposed method (based on the generative attention mechanism and generative state transition equation) in learning behavior analysis. The experimental results in Table 6 show that the proposed method outperforms the comparison models, including BERT, LLAMA, RoBERTa, GPT-3, XLNet, T5, and ELECTRA, across four metrics: accuracy, precision, recall, and F1-score. These results demonstrate the strong adaptability of the proposed method to complex learning behavior prediction tasks and highlight the significant improvements brought by its generative features. The experimental design uses a unified dataset and evaluation criteria to ensure the fairness and comparability of the results, thereby providing a reliable basis for the comprehensive evaluation of model performance.

### 4.3. Learning Experience Satisfaction Results

The purpose of this experiment is to compare the performance of different models in terms of learning experience satisfaction and to validate the proposed method’s advantages in enhancing student engagement and satisfaction. The experiment evaluates multiple dimensions, including satisfaction scores, standard deviation, positive feedback ratio, and negative feedback ratio, to comprehensively assess the effectiveness of each model in improving the learning experience. The results in Table 7 demonstrate that the proposed method achieves the best performance across all evaluation metrics, with a mean satisfaction score of 89.2, a standard deviation of 2.8, a positive feedback ratio of 94.3%, and a negative feedback ratio of only 5.7%. In contrast, models like GPT-3 and T5, which are relatively strong performers, achieved satisfaction scores of 81.5 and 82.4, with positive feedback ratios of 87.3% and 88.5%, respectively. Traditional models like BERT showed comparatively lower satisfaction scores at 76.5, with a positive feedback ratio of 82.1%. These results strongly confirm the proposed method’s significant advantages in enhancing the learning experience.

### 4.4. Sentiment Analysis and Behavioral Dynamics Capture Results

The primary objective of this experiment is to compare the performance of multiple models in sentiment analysis and behavioral dynamics capture tasks, validating the superiority of the proposed method in predicting emotional states and analyzing learning behaviors. The experiment focuses on four core evaluation metrics—sentiment prediction accuracy, behavioral recall, behavioral precision, and F1-score—to comprehensively assess each model’s performance in multitask scenarios. The results in Table 8 show that the proposed method achieved the highest scores across all metrics, with a sentiment prediction accuracy of 90.6%, a behavioral recall of 88.4%, a precision of 89.3%, and a combined F1-score of 88.8%. In contrast, top-performing models such as T5 and GPT-3 scored 86.4% and 85.7% in sentiment prediction accuracy, respectively, while their behavioral recall and precision metrics were also lower than those of the proposed method. Traditional models like BERT showed relatively lower overall performance, achieving only 78.5% in sentiment prediction accuracy. These findings strongly demonstrate the significant advantages of the proposed generative attention mechanism and the generative state transition equation in the joint modeling of sentiment and behavior.

## 5. Discussion

### 5.1. Behavior Prediction Results Discussion

From the experimental results, it is evident that traditional pretrained models like BERT and RoBERTa perform steadily in behavior prediction based on sequence relationships, but their reliance on static attention mechanisms limits their ability to adapt to temporal variations in student behaviors. XLNet and GPT-3 demonstrate stronger performance in long-term dependency modeling, capturing more contextual relationships within student behavior sequences. However, GPT-3’s reliance on fixed probabilistic generation mechanisms introduces limitations in modeling dynamic emotional fluctuations, as it does not inherently adjust its attention weights based on real-time behavioral transitions. Additionally, GPT-3’s large-scale generative architecture, while powerful in open-ended text generation, lacks fine-grained adaptation to structured behavior prediction tasks, where specific temporal dependencies play a crucial role. ELECTRA, as a lightweight model, performs well in low-resource scenarios but struggles with more complex, multimodal behavioral dynamics. In contrast, the proposed generative attention mechanism, combined with the generative state transition equation, dynamically generates attention weights based on historical behavior data while modeling emotional states with fine-grained precision. This approach outperforms GPT-3 and other baselines because it explicitly embeds temporal dependencies into the state transition equation, allowing the model to adjust to real-time changes in behavior patterns. Moreover, the generative loss function enables joint optimization of behavior and sentiment prediction, which GPT-3 lacks due to its independent probabilistic sequence generation mechanism. This dynamic adaptability improves prediction accuracy, enhances the model’s ability to track behavioral trends, and provides stronger technical support for personalized interventions.

### 5.2. Learning Experience Satisfaction Results Discussion

From a theoretical perspective, pretrained models like BERT and RoBERTa, which rely on static attention mechanisms, fail to dynamically adapt to students’ emotional changes, making it difficult to provide personalized support during learning. This contributes to their lower satisfaction scores, as they lack real-time adaptability in interactive learning scenarios. Generative models like GPT-3 and T5 exhibit stronger contextual understanding and text generation capabilities, which provide some level of interaction and personalized feedback. However, these models are still limited in dynamic learning environments, particularly in capturing emotional fluctuations and predicting complex behavioral trajectories.

The proposed generative attention mechanism, in combination with the generative state transition equation, dynamically adjusts attention weights and learning state transitions over time, allowing for a comprehensive capture of student behavior and emotions. Unlike GPT-3, which generates responses based on token probability distributions without intrinsic emotional state modeling, our model integrates behavioral dynamics directly into the learning process, ensuring a more context-aware adaptation. Furthermore, the introduction of the generative loss function facilitates a synergistic balance between behavioral guidance and emotional support, significantly improving engagement and learning satisfaction. This adaptive learning design not only increases students’ interest in the content but also provides a more tailored, emotionally responsive learning experience, surpassing other models in satisfaction ratings.

### 5.3. Sentiment Analysis and Behavioral Dynamics Capture Results Discussion

From a theoretical perspective, these results are closely related to the structural characteristics of different models. As representatives of pretrained models, BERT and RoBERTa perform consistently in static text modeling tasks but struggle to capture the dynamic changes in students’ emotional states and the complex temporal characteristics of behaviors due to their static attention mechanisms. In contrast, XLNet and GPT-3 introduce more flexible contextual modeling capabilities, achieving higher scores in behavioral dynamics capture and sentiment analysis but lacking the ability to dynamically optimize personalized learning paths during the generative process. The T5 model employs a unified text-to-text generation framework for multitask optimization, significantly improving the comprehensive capability for behavioral and sentiment analysis, though it still has limitations in handling long-term dependencies and dynamic transitions.

The proposed generative attention mechanism overcomes these challenges by dynamically adjusting attention weights and optimizing state transitions to fully capture students’ behavioral patterns and emotional fluctuations. Unlike GPT-3, which relies on static generative probabilities, our model’s generative state transition equation introduces a more context-aware adaptation by modeling the underlying temporal dependencies of student behaviors. Moreover, the generative loss function achieves joint optimization of sentiment and behavior prediction, leading to more accurate sentiment tracking and personalized learning path generation.

In particular, GPT-3’s lack of explicit state modeling mechanisms makes it less effective in tasks that require continuous adaptation to real-time behavioral inputs. While GPT-3 can generate coherent and contextually relevant outputs, its inability to dynamically regulate attention weights in response to changing student behaviors and emotions results in weaker performance in behavioral prediction. In contrast, the proposed model explicitly integrates temporal dependencies and state-driven adjustments, ensuring a superior capacity for behavioral dynamics modeling.

### 5.4. Failure Case Analysis

While the proposed generative attention mechanism demonstrates superior performance in behavior prediction, emotional state modeling, and adaptive learning interventions, certain failure cases highlight its limitations. One notable challenge arises in low-frequency behavioral patterns, where the model tends to overfit high-frequency interactions in the training data, leading to reduced accuracy when predicting rare or anomalous student behaviors. Additionally, in long-term sequential dependencies, despite the model’s dynamic attention adjustment, it struggles to retain distant behavioral patterns, occasionally resulting in inaccurate predictions for students with inconsistent engagement trends. Another observed limitation is in extreme emotional state classifications, where the model, primarily relying on behavioral and textual cues, sometimes misclassifies students’ emotions due to the absence of multimodal data, such as facial expressions or vocal intonations. These failure cases suggest potential directions for future improvements, including data augmentation for rare behaviors, enhanced memory mechanisms for long-term dependencies, and integration of multimodal inputs for more robust emotion recognition.

### 5.5. Limitations and Potential Risks of the Proposed Approach

Despite the advantages of the proposed generative attention mechanism and generative state transition equation, several limitations and potential risks must be acknowledged. One key concern is overfitting and data bias. Since the model learns from historical student behavior data, it may develop biases toward dominant behavior patterns in the dataset, leading to reduced generalization across diverse student populations. Additionally, sentiment prediction accuracy may be affected by incomplete or imbalanced emotional expression data, particularly if cultural or contextual variations in student emotions are not adequately represented. Another challenge is the lack of explainability in generative AI models, as the dynamic adjustment of attention weights and state transitions can make decision-making processes opaque, reducing trust and interpretability for educators. Furthermore, while the model adapts in real-time, unexpected behaviors or emotional shifts may not be captured effectively, potentially limiting its responsiveness in highly dynamic learning environments. Future research should explore fairness-aware training strategies, explainable AI techniques, and multimodal emotion modeling to enhance the robustness and applicability of the proposed approach.

### 5.6. Future Work

This paper proposes a method based on generative attention mechanisms and spatial state transition equations, demonstrating significant advantages in student behavior prediction and sentiment analysis. The model excels in capturing emotional state fluctuations and learning behavior dynamics, outperforming traditional methods such as BERT and LLAMA in handling the complexity of student behaviors during the learning process. The proposed approach effectively models emotional transitions and personalized learning states, making it particularly useful for adaptive education systems. Although the method performs exceptionally well across multiple evaluation metrics, its training and inference processes remain computationally complex, especially when dealing with large-scale datasets. Future research could focus on optimizing the model architecture to enhance its computational efficiency and scalability. Additionally, integrating sensor data (e.g., biometric feedback, learning device interactions) and leveraging advanced generative models (e.g., GANs, Transformers) could further improve prediction accuracy and real-time adaptation capabilities.

Moreover, while the current evaluation relies on quantitative metrics such as accuracy, recall, and F1-score, we also collected real teacher feedback to assess the model’s practical effectiveness. For instance, in a classroom case study, a teacher observed that the model successfully identified a student with low engagement and emotional fluctuations, aligning with the teacher’s own assessment. However, due to the limited sample size, we primarily focused on quantitative analysis in this study. In future work, we plan to conduct larger-scale experiments, incorporating more extensive teacher feedback and qualitative observations to further validate the model’s alignment with real-world teaching scenarios. Expanding the model’s applications across diverse educational settings will also help assess its generalizability and practical impact.

## 6. Conclusions

This study explores the application of digital cultural resources in Chinese language education, proposing a learning behavior analysis method based on the generative attention mechanism and generative state transition equation. The method addresses the limitations of traditional approaches in capturing dynamic behavioral patterns and emotional states. By integrating deep learning models with educational applications, this study introduces a generative loss function, enabling joint optimization of sentiment prediction and behavior analysis. Experimental results demonstrate that the proposed model outperforms existing benchmark models across multiple evaluation metrics, achieving 90.6% accuracy, 88.4% recall, 89.3% precision, and an F1-score of 88.8% in behavior prediction tasks. Additionally, it achieves a learning satisfaction score of 89.2, with a 94.3% positive feedback rate, surpassing models such as BERT, GPT-3, and T5. These findings validate the practical effectiveness of the method in enhancing learning engagement and personalized teaching strategies.

Despite its promising results, this study has several limitations. The current study primarily focuses on behavioral and sentiment analysis without integrating multimodal learning signals such as speech tone, facial expressions, and physiological indicators, which could provide a more comprehensive understanding of student engagement. Moreover, while the model demonstrates strong predictive performance, its practical deployment in real-world educational settings requires further validation, particularly in adaptive learning platforms and large-scale classroom environments.

To further expand this research, several directions can be pursued. Future studies should explore multimodal data fusion to enhance the accuracy of behavioral and emotional state modeling. Additionally, integrating reinforcement learning techniques could enable more adaptive and personalized learning pathways, improving real-time intervention strategies. In summary, this research provides a novel framework for dynamic behavior analysis and sentiment modeling, offering both theoretical contributions and practical insights into personalized teaching interventions in Chinese language education. Future advancements in AI-driven educational technologies can further optimize adaptive learning strategies, ultimately enhancing student engagement and academic performance.

## Figures and Tables

**Table 1:** Pretest Score Normality Test Results.

Group	Sample Size	Mean	Std Dev	Skewness	Kurtosis	Kolmogorov–Smirnov Test		Shapiro–Wilk Test	
						Statistic	*p*-Value	Statistic	*p*-Value
A	7	35.143	2.968	0.556	−0.716	0.221	0.376	0.907	0.374
B	7	35.143	4.880	−0.374	0.313	0.122	0.989	0.990	0.993

**Table 2:** Pretest Score *t*-Test Results.

Content	Group (Mean ± Std Dev)		t-Statistic	*p*-Value
	Group A	Group B		
Predicted Performance	35.14 ± 2.97	35.14 ± 4.88	0.000	1.000

**Table 3:** Normality Test Results for Three Types of Knowledge Scores.

Group	Mean	Std Dev	Skewness	Kurtosis	Kolmogorov–Smirnov Test		Shapiro–Wilk Test	
					Statistic	*p*-Value	Statistic	*p*-Value
Bronze and Porcelain Knowledge								
A	9.714	2.138	−0.772	0.263	0.267	0.136	0.894	0.294
B	8.571	2.225	0.249	−0.944	0.173	0.762	0.992	0.482
Bronze Craftsmanship Topic								
A	15.143	2.116	−1.442	2.080	0.229	0.326	0.854	0.133
B	17.571	1.618	−0.674	−1.151	0.240	0.258	0.864	0.163
Porcelain Evaluation Topic								
A	16.714	1.380	0.706	−0.325	0.269	0.103	0.918	0.456
B	14.429	2.070	−0.489	−0.361	0.205	0.503	0.945	0.686

**Table 4:** *t*-Test Results for Three Types of Knowledge Scores.

Content	Group (Mean ± Std Dev)		t-Statistic	*p*-Value
	Group A	Group B		
Basic Knowledge	9.714 ± 2.138	8.571 ± 2.225	0.980	0.347
Bronze Knowledge	15.143±2.116	17.571±1.618	−2.412	0.033
Porcelain Knowledge	16.714±1.380	14.429±2.070	2.431	0.032

**Table 5:** Interview information.

Interviewee	Experimental Group	Gender	Interview Date	Interview Location
Interviewee 1	Group A	Male	15 January 2024 16:00	Tencent Meeting 207-219-815
Interviewee 2	Group A	Male	16 January 2024 9:30	Tencent Meeting 748-925-593
Interviewee 3	Group B	Female	15 January 2024 20:00	Tencent Meeting 599-892-029
Interviewee 4	Group B	Female	16 January 2024 20:00	Tencent Meeting 565-305-123

**Table 6:** Behavior Prediction Results Comparison.

Model	Accuracy (%)	Precision (%)	Recall (%)	F1-Score (%)
BERT	82.4	81.8	80.5	81.1
LLAMA	84.2	83.7	82.9	83.3
RoBERTa	85.6	85.0	84.3	84.6
GPT-3	87.8	87.0	86.4	86.7
XLNet	86.5	85.9	85.1	85.5
T5	88.1	87.5	86.9	87.2
ELECTRA	85.9	85.2	84.6	84.9
Self-Attention	88.9	88.0	88.3	88.4
CBAM Attention	79.3	80.1	80.2	79.8
Proposed Method	90.3	89.7	89.0	89.4

**Table 7:** Learning Experience Satisfaction Results Comparison.

Model	Satisfaction Score	Std. Deviation	Positive Feedback (%)	Negative Feedback (%)
BERT	76.5	5.4	82.1	17.9
LLAMA	78.3	4.8	83.5	16.5
RoBERTa	79.8	4.2	85.7	14.3
GPT-3	81.5	3.9	87.3	12.7
XLNet	80.7	4.0	86.9	13.1
T5	82.4	3.6	88.5	11.5
ELECTRA	79.1	4.5	85.2	14.8
Self-Attention	84.3	3.1	89.6	12.0
CBAM Attention	80.4	4.5	83.7	13.2
Proposed Method	89.2	2.8	94.3	5.7

**Table 8:** Sentiment Analysis and Behavioral Dynamics Capture Results Comparison.

Model	Sentiment Accuracy (%)	Behavioral Recall (%)	Behavioral Precision (%)	F1-Score (%)
BERT	78.5	75.4	76.2	75.8
LLAMA	81.3	78.5	79.1	78.8
RoBERTa	83.2	80.6	81.0	80.8
GPT-3	85.7	83.1	84.3	83.7
XLNet	84.5	82.7	83.5	83.1
T5	86.4	84.8	85.5	85.1
ELECTRA	82.9	80.3	81.2	80.7
Self-Attention	86.1	84.9	85.4	85.3
CBAM Attention	71.3	70.7	70.9	71.0
Proposed Method	90.6	88.4	89.3	88.8

## Data Availability

The data presented in this study are available oupon request from the corresponding author.
